# Boosting Ethanol Productivity of *Zymomonas mobilis* 8b in Enzymatic Hydrolysate of Dilute Acid and Ammonia Pretreated Corn Stover Through Medium Optimization, High Cell Density Fermentation and Cell Recycling

**DOI:** 10.3389/fmicb.2019.02316

**Published:** 2019-10-04

**Authors:** Ying Li, Rui Zhai, Xiaoxiao Jiang, Xiangxue Chen, Xinchuan Yuan, Zhihua Liu, Mingjie Jin

**Affiliations:** ^1^School of Environmental and Biological Engineering, Nanjing University of Science and Technology, Nanjing, China; ^2^Department of Plant Pathology and Microbiology, College of Agriculture and Life Sciences, Texas A&M University, College Station, TX, United States

**Keywords:** high cell density fermentation, cell recycling, *Zymomonas mobilis* 8b, hydrolysate, ethanol productivity

## Abstract

The presence of toxic degradation products in lignocellulosic hydrolysate typically reduced fermentation rates and xylose consumption rate, resulting in a decreased ethanol productivity. In the present study, *Zymomonas mobilis* 8b was investigated for high cell density fermentation with cell recycling to improve the ethanol productivity in lignocellulosic hydrolysate. The fermentation performances of *Z. mobilis* 8b at various conditions were first studied in yeast extract-tryptone medium. It was found that nutrient level was essential for glucose and xylose co-fermentation by *Z. mobilis* 8b and high cell density fermentation with cell recycling worked well in yeast extract-tryptone medium for 6 rounds fermentation. *Z. mobilis* 8b was then studied in enzymatic hydrolysates derived from dilute acid (DA) pretreated corn stover (CS) and ammonia pretreated CS for high cell density fermentation with cell recycling. Ethanol productivity obtained was around three times higher compared to traditional fermentation. Ethanol titer and metabolic yield were also enhanced with high cell density fermentation. *Z. mobilis* 8b cells showed high recyclability in ammonia pretreated CS hydrolysate.

## Introduction

Bioconversion of lignocellulosic biomass to ethanol has drawn great attention because of its benefit to environmental, economic, and social sustainability. However, the high production cost of lignocellulosic ethanol remains the bottleneck for its commercialization (Jin et al., 2012; Karagoz et al., 2019). One of the causes for the high production costs is the low volumetric productivity (Ferreira et al., 2018). Although many previous studies have engineered the microorganisms to improve the fermentation rates of glucose and xylose, the overall xylose fermentation rate remains slow (Ong et al., 2018; Yang S. et al., 2018; Xia et al., 2019). On the other hand, process engineering could be used to enhance the volumetric productivity (Matano et al., 2013; Zhou et al., 2017). As reported by our previous study, high cell density fermentation coupled with cell recycling increased cellulosic ethanol productivity by twofold–threefold (Jin et al., 2012, 2016). However, our previous studies focused on the improvement of fermentation efficiencies of *Saccharomyces cerevisiae*, while other commonly used fermenting microorganisms were not assessed. Compared with *S. cerevisiae*, *Zymomonas mobilis* 8b has been reported to exhibit excellent ethanol productivity, high ethanol tolerance and efficient sugar uptake (Rogers et al., 2007; Clarke et al., 2018; Xia et al., 2019). Recently, many studies have engineered *Z. mobilis* strains to enable it to utilize both five-carbon and six-carbon sugars (Yang S. et al., 2018; Yang Y. et al., 2018; Yang et al., 2019). Although the molecular biology and fermentation capabilities of these *Z. mobilis* recombinants have been extensively documented, the reduced volumetric productivity of these recombinants in lignocellulosic hydrolysate was also observed due to the presence of toxic degradation products (Zheng et al., 2019). High cell density has been used for ethanol fermentation as it can significantly accelerate fermentation rates, eliminate unproductive lag phase and promote inhibitor tolerance (Sarks et al., 2014). In addition, cell recycling can serve as useful strategy to maintain sufficient cell density and efficient fermentation. We have demonstrated high cell density fermentation with cell recycling on *S. cerevisiae* (Jin et al., 2012, 2016) and preliminary found the great potential of this strategy on *Z. mobilis* 8b. Thus, it would be interesting to further study and demonstrate the possibility of using high cell density fermentation strategy to improve the ethanol productivity of *Z. mobilis* 8b in lignocellulosic hydrolysate.

To obtain sugar streams from lignocellulosic biomass for fermentation, a pretreatment is needed to break up the lignocellulose and enzymatic hydrolysis is required to degrade the polysaccharide into fermentable monosaccharides, such as glucose and xylose (Lai et al., 2019). Many pretreatment strategies such as dilute acid (DA) (Zhai et al., 2018; Huang et al., 2019), dilute alkali (McIntosh and Vancov, 2011), steam explosion (Liu and Chen, 2017; Zhong et al., 2019), liquid hot water (Zhuang et al., 2016; Tian et al., 2019), ammonia fiber expansion (Serate et al., 2015; Jin et al., 2016), and extractive ammonia pretreatment (Sousa et al., 2016) have been developed to deconstruct the lignocellulose to improve their hydrolysablity by cellulases. Among these pretreatment, DA pretreatment has been studied widely because of the advantages such as simple operation, easy industrial application, and hemicellulose degradation (Alvira et al., 2010; Pedersen et al., 2011). However, DA pretreatment generates toxic compounds that impair fermentation (Zhai et al., 2016; Liu et al., 2018). Different from DA pretreatment, ammonia pretreatment was featured with its low toxicity toward fermentation as well as capability to convert cellulose I to cellulose III (Wada et al., 2006; Ong et al., 2016). As these two promising pretreatments have been studied widely for the commercial production of ethanol from lignocellulosic biomass, they could serve as representative pretreatment methods.

In the present study, we systematically studied the fermentation behaviors of *Z. mobilis* 8b and identified the essential conditions that affect the fermentation rate and ethanol productivity. Based on the fermentation performances of *Z. mobilis* 8b, we designed and investigated high cell density fermentation with cell recycling in both synthetic medium and lignocellulosic hydrolysate derived from DA and ammonia pretreated corn stover (CS).

## Materials and Methods

### Materials

Corn stover was harvested at Lianyungang, Jiangsu, China. The contents of glucan, xylan, lignin, and ash were determined with the standard method published by National Renewable Energy Laboratory (NREL) (Sluiter et al., 2008). Cellulase (60 mg protein/mL, 118 FPU/mL) and xylanase (25 mg protein/mL, 100,000 U/mL for Beachwood xylan) were provided by Qingdao Vland Biotech Inc.

### Pretreatment

Dilute acid and ammonia pretreatments were performed according to previous studies with a little modification (Hsu et al., 2010; Da Costa Sousa et al., 2016). Specifically, for DA pretreatment, CS was pretreated in a 2 L high pressure reactor (Weihai Chemical Device, Shandong, China) with 10% (w/w) solid loading and 1% w/w H_2_SO_4_, at 160°C for 10 min. After pretreatment, the CS slurry was neutralized by 10 M potassium hydroxide to pH 7, and dried at 60°C until the moisture of treated CS was between 10 and 20%. The DA pretreated CS was composed of 27.2% glucan and 12.7% xylan. Ammonia pretreatment was similar with extractive ammonia pretreatment except that the extraction step was removed. Specifically, the ammonia pretreatment was carried out by soaking 10% (w/w) CS (dry weight basis) in anhydrous liquid ammonia (6 g ammonia/g dry biomass) at 120°C for 30 min. The sample was dried in fume hood overnight to remove residual ammonia. The ammonia pretreated CS was composed of 31.6% glucan and 17.8% xylan. All the pretreated CS was stored at 4°C.

### Enzymatic Hydrolysis of Pretreated CS

DA-CS (dilute sulfuric acid pretreated CS) and A-CS (ammonia pretreated CS) were hydrolyzed using commercial enzyme mixture including cellulase (28 mg protein/g glucan) and xylanase (12 mg protein/g glucan). Enzymatic hydrolysis was carried out at 18% (w/w) solid loading in 250 mL flasks at pH 4.8, 50°C and 250 rpm. After 72 h hydrolysis, the enzymatic hydrolysate was centrifuged at 10,000 rpm for 10 min. Supernatant’s pH was adjusted by 10 M potassium hydroxide to pH 5.8, and then kept in a sterile bottle. Hydrolysate obtained from DA at 18% solid loading contained 47.7 g/L glucose and 28.3 g/L xylose, while hydrolysate obtained from ammonia pretreated CS contained 65.8 g/L glucose and 22.9 g/L xylose.

### Microorganism and Seed Culture Preparation

For *Z. mobilis* 8b, a two-stage seed culture was applied. Pre-culture medium contained (g/L): 10 yeast extract (YE), 20 tryptone, 2 potassium dihydrogen phosphate, 50 glucose, 20 xylose. A glycerol stock of *Z. mobilis* 8b was used to inoculate the medium. Seed culture was prepared in a 50 mL flask with 40 mL medium at 30°C and 150 rpm. When the OD_600_ reached around 6, 5 mL of the culture was then transferred to another fresh pre-culture medium. When the OD_600_ of the pre-culture reached around 10, the cells were centrifuged and transferred to fermentation medium.

### Fermentation in Yeast Extract-Peptone Medium or Hydrolysate

Fermentation in yeast extract-peptone (YP) medium or in hydrolysate was conducted at 150 rpm, 30°C in 50 mL flasks with a working volume of 40 mL. The concentration of each component in YP medium were 1–10 g/L for YE, 2–20 g/L for tryptone, 60–120 g/L for glucose (G), 30–90 g/L for xylose (X). For hydrolysate fermentation, 6 M KOH was used to adjust the pH of the enzymatic hydrolysate to 5.8. To release carbon dioxide generated during fermentation, rubber stoppers pierced through by a hollow needle were used to cap the flasks. Fermentation was performed in triplicate and means and standard deviations were calculated. The significance of difference were further analyzed using the one-way ANOVA test.

### High Cell Density Fermentation With Cell Recycling

The fermentation was performed in a 50 mL flask with working volume of 40 mL at 30°C, 150 rpm. *Z. mobilis 8b* cell obtained from centrifugation of seed culture was used for inoculation to reach an initial OD_600_ of 1 and 8. After 24 h fermentation, the cell was separated by centrifugation at 5,000 rpm for 10 min. Then, the recycled cell was resuspended to fresh hydrolysate for the next round fermentation.

### Analysis Methods

Sugars and ethanol were analyzed by high-performance liquid chromatography (HPLC) using refractive index detector and a Biorad Aminex HPX-87H column at 65°C with 5 mM sulfuric acid as the mobile carrier at a flow rate 0.6 mL/min. Each sample was diluted and filtered through a 0.22 μm nylon syringe filter before analysis. Metabolic yield was calculated based on the theoretical ethanol yield from consumed glucose and xylose, which is 2 mol ethanol/mol glucose or 0.51 g ethanol/g glucose (1.67 mol/mol xylose or 0.51 g/g xylose). The metabolic yield, sugar conversion and ethanol productivity were calculated by Eqs 1–3, respectively:

(1) $$\mathrm{Metabolic}\,\mathrm{yield}=\frac{\mathrm{ethanol}\,\left(g\right)}{\text{sugar}\,\left(\text{g}\right)\times 0.51}\times 100\%$$

(2) $$\mathrm{Sugar}\,\mathrm{conversion}=\frac{{\text{sugar}}_{0}\,\left(g\right)-{\text{sugar}}_{\text{t}}\,\left(g\right)}{{\text{sugar}}_{0}\,\left(g\right)}\times 100\%$$

(3) $$\mathrm{Ethanol}\,\mathrm{productivity}=\frac{\mathrm{ethanol}\,\left(g\right)}{\mathrm{fermentation}\,\mathrm{volume}\,\left(\text{L}\right)\times \mathrm{fermentation}\,\mathrm{time}\,\left(h\right)}$$

where sugar*_t_* is the mass of sugar at fermentation time t.

## Results and Discussion

### Fermentation Characteristics of *Z. mobilis* 8b

#### Fermentation of *Z. mobilis* 8b on Glucose and Xylose

**FIGURE 1:**
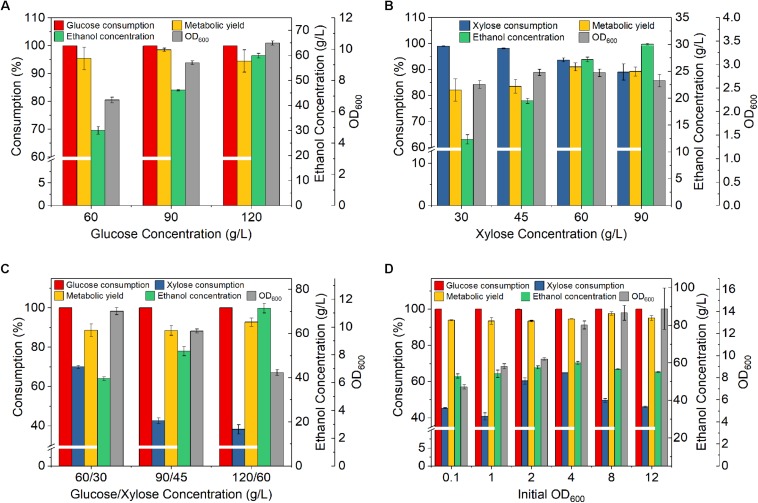
Effect of sugar concentrations and initial OD_600_ on fermentation by *Z. mobilis* 8b. **(A)** Fermentation with different glucose concentration; **(B)** fermentation with different xylose concentration; **(C)** fermentation with different concentrations of mixed sugars (glucose and xylose); and **(D)** fermentation with different initial OD_600_. The fermentation experiment was performed at 30°C, 150 rpm, for 72 h. For **(A–D)**, the nutrient concentrations were 2.5 g/L yeast extract (YE), 5 g/L tryptone; for **(A–C)**, the initial OD_600_ was 1, for **(D)**, sugar concentrations were 90 g/L glucose, 45 g/L xylose.

To improve the sugar utilization by *Z. mobilis* 8b and achieve satisfactory high cell density fermentation performance, understanding the factors that affect fermentation is necessary. Therefore, the effect of sugar concentrations on fermentation performance of *Z. mobilis* 8b was studied (Figure 1). The sugar concentrations were selected on the basis of sugar release from enzymatic hydrolysis of lignocellulosic biomass at different solid loadings. In general, *Z. mobilis* 8b grew significantly better in yeast extract-peptone-glucose (YPG) medium than in yeast extract-peptone-xylose (YPX) medium (one-way ANOVA test, *p* < 0.01). For example, the OD_600_ of the strain with 60 and 90 g/L glucose reached 6.7 and 9.1, respectively, which is much higher than that with the same concentrations of xylose (Figures 1A,B). This is consistent with the study reported by Kim et al. (2000). The reason for such result was likely due to the slower consumption of xylose, and less energy generated for cell growth during xylose metabolism.

For fermentation at different glucose concentrations, glucose was all completely consumed, and the metabolic yield of ethanol was all above 94.5%. This indicates that glucose concentration less than 120 g/L may have no significant effect on glucose consumption and ethanol yield (one-way ANOVA test, *p* > 0.05). However, the xylose consumption decreased gradually with the increase of xylose concentration, and dropped to 89% when xylose concentration reached 90 g/L (Figure 1B). This result was possibly caused by the increased ethanol concentration. Higher xylose concentration produced higher ethanol concentration, which in turn inhibited xylose fermentation and resulted in less xylose consumption. Furthermore, the metabolic yield on xylose (lower than 90% in most cases) was lower than that on glucose. Similar result was also observed by Yang et al. (2014). It has been suggested when only glucose was used as the carbon source, efficient *Z. mobilis* fermentation pathway enzymes allowed fast ethanol production with minimal acetate produced as byproducts. When only xylose was used as the carbon source (Yang et al., 2014), much higher concentration of toxic intermediates such as xylitol was produced, resulting in a inhibition of cell growth. Interestingly, the metabolic yields were higher with initial xylose concentrations of 60and 90 g/L as compared to lower ones. At 60 g/L xylose, the metabolic yield achieved 91%. This interesting phenomenon was unexpected. Xylose utilization could pose a significant metabolic burden to the cells of *Z. mobilis* 8b and trigger responses with gene expression (Yang et al., 2014). Different concentrations of xylose and ethanol might have induced different metabolic responses and hence led to different metabolic yields.

As both glucose and xylose are present in lignocellulosic hydrolysate, we further studied the performance of *Z. mobilis* 8b in yeast extract-peptone-glucose-xylose (YPGX) medium with different concentrations of glucose and xylose. Glucose was all completely consumed in all cases. However, xylose consumption was significantly reduced in the presence of glucose, compared to that using xylose as the sole carbon source (one-way ANOVA test, *p* < 0.05). This result was probably due to the presence of high ethanol concentration generated from glucose fermentation, which in turn inhibited xylose consumption. In addition, inefficient xylose transport represents another bottleneck. In *Z. mobilis*, xylose enters the cell through the diffusion protein that may be blocked by glucose through a competitive inhibition mechanism (Wang et al., 2018).

When the total sugar concentration increased to 180 g/L (120 g/L glucose and 60 g/L xylose), the xylose consumption decreased to 38% (Figure 1C). In addition, the OD was also significantly decreased with increasing mixed sugar concentration, which indicated that the growth of *Z. mobilis* 8b was greatly suppressed (one-way ANOVA test, *p* < 0.01). Hence, the concentrations of the mixed sugar less than or equal to 90 g/L glucose and 45 g/L xylose were selected for the following studies.

In order to improve the fermentation performance with mixed sugar, the effect of inoculation size was further studied. The xylose consumption increased to the highest of 64.8% when the initial OD increased to 4.0 (Figure 1D). Meanwhile, the metabolic yield was as high as 97.6%, which was higher than those using glucose or xylose as the sole carbon source. Further increasing initial OD did not further improve the fermentation performance. Rather, the xylose consumption was decreased. Overall, the mixed sugar fermentation was still not satisfactory. It was likely that a reasonable high OD was needed for mixed sugar fermentation. The fundamentals underlying this phenomenon may be quorum sensing, which has been reported as typical feature of Gram-negative bacteria such as *Escherichia coli* and *Pseudomonas aeruginosa* (Papenfort and Bassler, 2016). It is likely that the high cell density can trigger quorum sensing and help the cell to maintain favorable physiological trait that is important for bioethanol production. However, the nutrient level in the medium was not sufficient to sustain the cell activities at high cell density.

#### Effect of Nutrient Concentration on *Z. mobilis* 8b Fermentation

**FIGURE 2:**
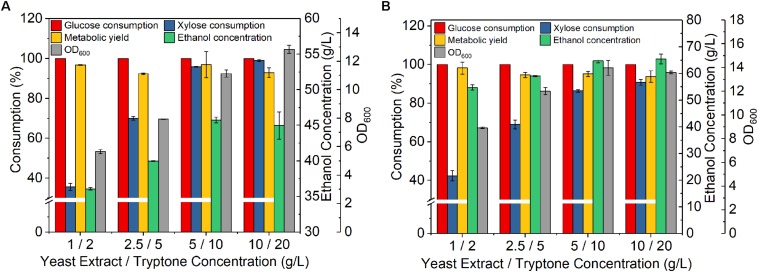
Effect of nitrogen nutrient concentrations on fermentation of *Z. mobilis* 8b in synthetic medium. The fermentation experiment was performed at 30°C, 150 rpm for 72 h. **(A)** the initial OD_600_ was 1, while the sugar concentrations were 60 g/L glucose and 30 g/L xylose; and **(B)** the initial OD_600_ was 4, while the sugar concentrations were 90 g/L glucose, 45 g/L xylose.

Nutrient has great impact on sugar utilization as the ability of strains to achieve a high level of ethanol titer greatly depends on the nutritional conditions and protective functions provided by some nutrients (Lau and Dale, 2009; Sarks et al., 2014). Thus, to further improve sugar utilization, the effect of nutrient (yeast extract and tryptone) concentration on fermentation of mixed sugars was studied. At 60 g/L glucose and 30 g/L xylose with initial OD 1, the increase of nutrient concentration improve both cell growth (OD) and xylose consumption (Figure 2A). With yeast extract 5 g/L and tryptone 10 g/L, the xylose consumption reached as high as 95.8% and ethanol concentration reach 45.7 g/L with a metabolic yield of 96.9% (Figure 2A). Although further increase of nutrient concentration improved xylose consumption, the increase was less obvious. At 90 g/L glucose and 45 g/L xylose with initial OD 4, the trend with increasing nutrient level was similar (Figure 2B). Therefore, the nutrient level 5 g/L yeast extract and 10 g/L tryptone seemed sufficient for mixed sugar fermentation.

**FIGURE 3:**
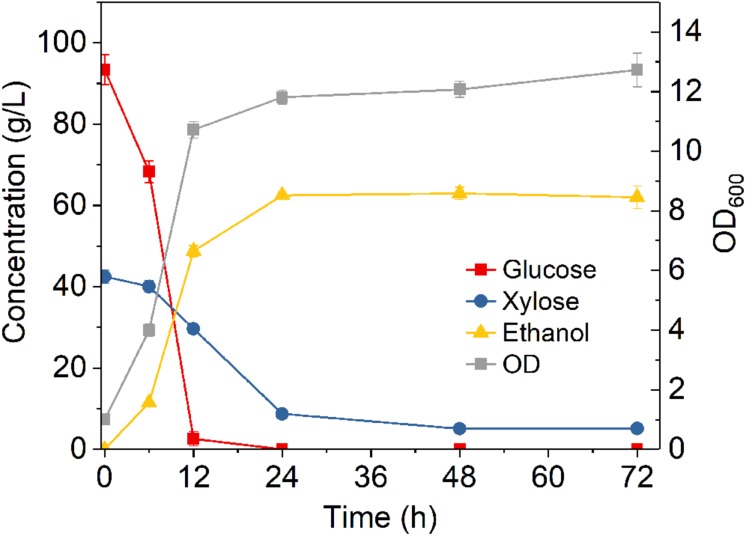
Fermentation time course of *Z. mobilis* 8b in yeast extract-peptone-glucose-xylose medium. The fermentation experiment was performed at 30°C, 150 rpm with initial OD_600_ of 1. The nutrient include 5 g/L yeast extract and 10 g/L tryptone.

After studying the effect of various conditions on fermentation, the sugar utilization and ethanol production kinetics under the optimized conditions were further investigated. As shown in Figure 3, glucose was rapidly and completely converted to ethanol within 12 h, while xylose was consumed much slower, with 8.7 g/L left unconsumed after 24 h fermentation. After 72 h fermentation, 5.1 g/L xylose remained unconsumed, accounting for a xylose consumption of 88%, while the ethanol titer reached 63 g/L, accounting for a metabolic yield of 94.4%. The fact that xylose consumption was also greatly improved suggested that sufficient nutrients are needed for fully support high cell population for ethanol fermentation. In addition, as *Z. mobilis* 8b is an engineered strain that requires heterogenous expression of enzymes for the conversion of xylose into ethanol, it is thus expected that higher nitrogen level facilitated the overexpression of certain enzymes that promote the xylose consumption.

### Cell Recycling of *Z. mobilis* 8b for Fermentation on Mixed Sugars

**FIGURE 4:**
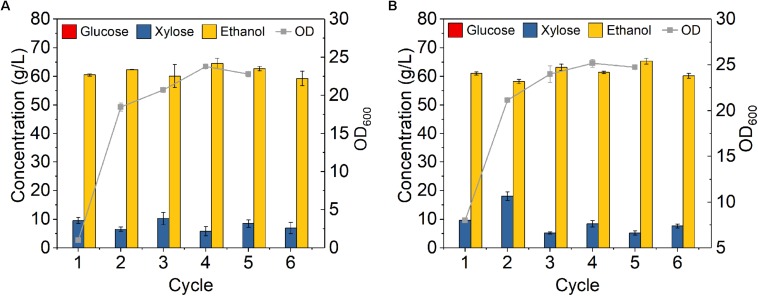
Cell recycling with 75% cell recycled after 24 h fermentation on yeast extract-peptone medium using *Z. mobilis 8b* with **(A)** initial OD_600_ of 1 and **(B)** initial OD_600_ of 8. The fermentation experiment was performed at 30°C, 150 rpm. The nutrient includes 5 g/L yeast extract and 10 g/L tryptone. The sugar mixture includes 90 g/L glucose and 45 g/L xylose. Cell recycling was performed after 24 h fermentation, and for each cycle, 75% of cell was recycled for next round of fermentation.

To evaluate the feasibility of high cell density fermentation with cell recycling using *Z. mobilis* 8b, 6-cycle fermentations were carried out on YPGX medium with initial ODs of 1 and 8, respectively. As cell recycling led to accumulation of large amount of cells in the reactor, it might be too much for fermentation and might cause activity loss of partial cells (Sarks et al., 2014). Thus, instead of recycling all cells, 75% cells were recycled for each 24-h fermentation cycle. As shown in Figure 4, the fermentation performance did not decrease from cycle to cycle. With initial ODs of 1 (Figure 4A) and 8 (Figure 4B), glucose was completely consumed at the end of each cycle, leaving less than 10 g/L xylose in the fermentation broth. The overall ethanol productivity of each cycle was kept between 2.4 and 2.6 g L^–1^ h^–1^ after six cycles (Figure 4). This was higher than the study reported by Kim et al., who used *Z. mobilis* 8b for fermentation at sugar concentration of 100 g/L and obtained 1.43 g L^–1^ h^–1^ (Clarke et al., 2018). The increased ethanol productivity was related with the high cell density fermentation with cell recycling, which can accelerate fermentation in the reactor (Santos et al., 2016). Therefore, it seems high cell density fermentation with cell recycling worked well on *Z. mobilis* 8b.

### Fermentation of A-CS Hydrolysate by *Z. mobilis* 8b at High Cell Density

**FIGURE 5:**
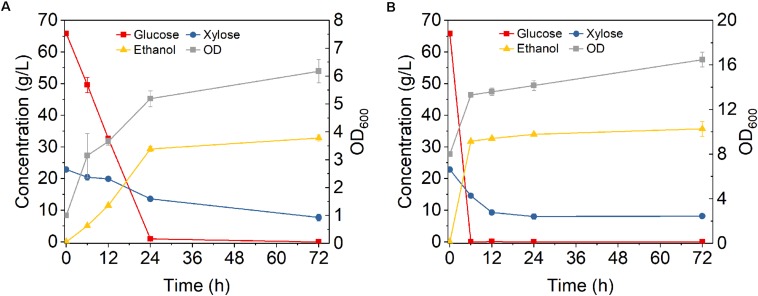
Fermentation performance of *Z. mobilis* 8b in hydrolysate derived from enzymatic hydrolysis of ammonia pretreated corn stover at 18% solid loading with **(A)** initial OD_600_ of 1 and **(B)** initial OD_600_ of 8. The fermentation experiment was performed at 30°C, 150 rpm. Nutrients supplemented include 5 g/L yeast extract and 10 g/L tryptone.

**FIGURE 6:**
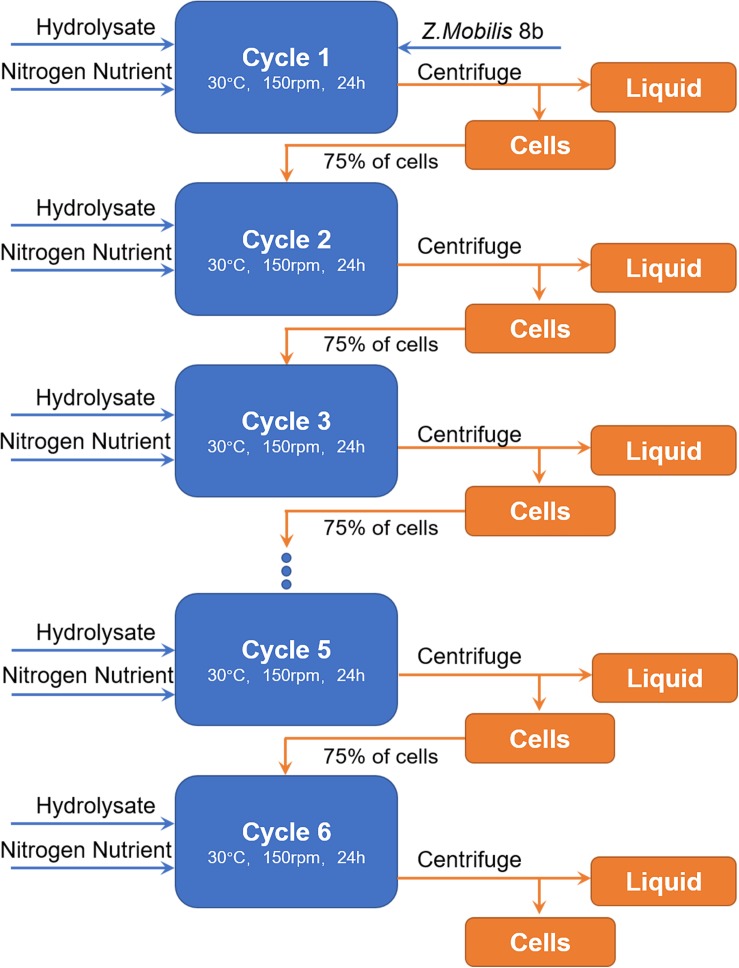
Flow chart of cell recycling strategy. In each cycle, fresh hydrolysate was added in the reactor. Fresh cells were only added at the first round. From second to sixth round, 75% of cells in previous round were collected by centrifugation and recycled to the following cycle.

To investigate the feasibility of using high cell density fermentation with cell recycling in real lignocellulosic hydrolysate, A-CS and DA-CS were used for study. Fermentation of A-CS hydrolysate was first tested using *Z. mobilis* 8b with initial ODs of 1 and 8. With initial OD 1, glucose was not completely consumed until around 24 h (Figure 5A). The glucose consumption rate in A-CS hydrolysate was much slower than that in YP, as glucose was almost consumed after 12 h in YP (Figure 3). According to a previous study, 1 g ammonia pretreated CS could release around 651 μg furans, 1.7 mg aromatic compounds and 10 mg carboxylic acids (Chundawat et al., 2010). Thus, this result was likely due to the presence of toxic degradation products in the A-CS hydrolysate which reduced the fermentation performance. Furthermore, xylose consumption was also much slower than that in YP medium with 7.7 g/L xylose remained in the fermentation broth after 72 h. When the initial OD increased to 8, it only took 6 h to consume all the glucose in the hydrolysate and took around 24 h to reduce xylose concentration to the same level as that with initial OD 1 (Figure 5B). In addition, when the initial OD increased from 1 to 8, the metabolic yield increased from 79.5 to 87.0% and the final ethanol titer increased from 32.8 to 35.7 g/L. Therefore, high cell density fermentation with *Z. mobilis* 8b worked in A-CS hydrolysate, which greatly shortened the fermentation time and thus increased the productivity. Cell recycling with initial OD 8 for the first cycle was then performed (Figure 6).

**FIGURE 7:**
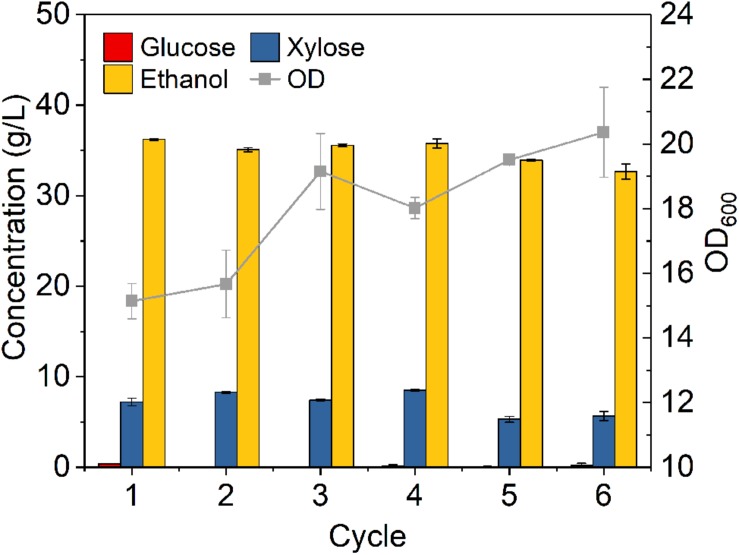
Cells recycling in fermentation of hydrolysate of ammonia pretreated corn stover. The fermentation experiment was performed at 30°C, 150 rpm with initial OD_600_ of 8. The nutrients supplemented included 5 g/L yeast extract and 10 g/L tryptone.

Figure 7 shows that glucose was completely consumed in each 24-h cycle, while unconsumed xylose concentration slightly fluctuated from cycle to cycle in the range of 5.3–8.5 g/L. Ethanol titer of each cycle was between 36.2 and 32.7 g/L with OD increased from cycle to cycle. Although there were some fluctuations, the overall fermentation performance was slightly reduced from cycle to cycle. High density fermentation and cell recycling produced metabolic yield of 87.8–94.5% and ethanol productivity of 1.70–1.89 g L^–1^ h^–1^, which showed superior performance as compared to traditional fermentation with metabolic yield of 79.5% and productivity of 0.46 g L^–1^ h^–1^ (Figure 5A).

### Fermentation of DA-CS Hydrolysate by *Z. mobilis* 8b at High Cell Density

**FIGURE 8:**
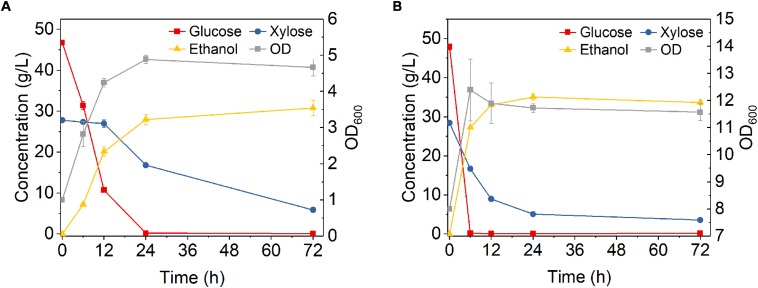
Fermentation performance of hydrolysate of dilute sulfuric acid pretreated corn stover by *Z. mobilis* 8b with different initial OD_600_. **(A)** initial OD_600_ was 1; **(B)** initial OD_600_ was 8. The fermentation experiment was performed at 30°C, 150 rpm for 72 h. The nutrients supplemented included 5 g/L yeast extract and 10 g/L tryptone.

Fermentation performance of *Z. mobilis* 8b in DA-CS hydrolysate was also studied. Similar to fermentation in A-CS hydrolysate, with initial OD 1, it took 24 h to consume all the glucose and took 72 h to reduce xylose concentration to 5.9 g/L, which was much longer than the fermentation in YP medium (Figure 8A). The final ethanol titer and metabolic yield was 30.8 g/L and 88.1%, respectively, which were lower than that in synthetic medium. When the initial OD increased to 8, glucose was consumed rapidly in the first 6 h and xylose was almost consumed within 24 h, reaching a final xylose concentration of 3.6 g/L (Figure 8B). The ethanol titer was 35.1 g/L, while the ethanol productivity and metabolic yield in the first 24 h was 1.82 g L^–1^ h^–1^ and 96.44%, respectively. These results were all higher as compared to that with initial OD 1.

**FIGURE 9:**
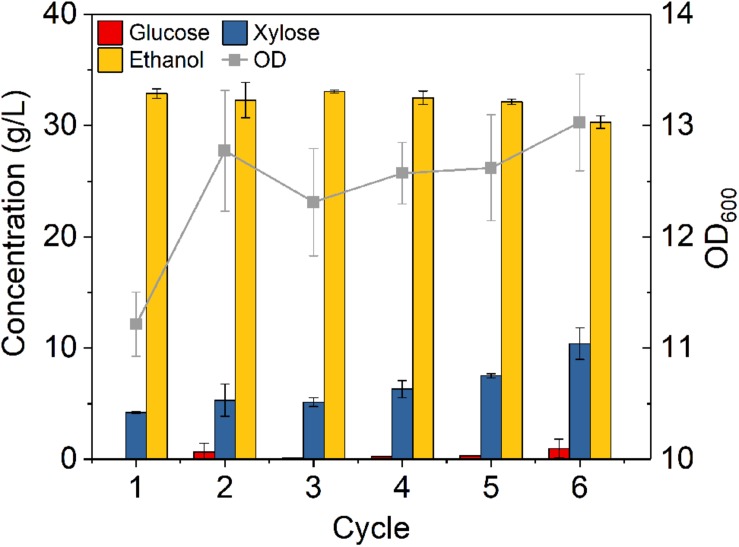
Cells recycling in fermentation of hydrolysate of dilute sulfuric acid pretreated corn stover. The fermentation experiment was performed at 30°C, 150 rpm with initial OD_600_ of 8. The nutrients supplemented included 5 g/L yeast extract and 10 g/L tryptone.

The fermentation performance of DA-CS hydrolysate with six round cell recycling was also studied at initial OD 8 for the first round fermentation. During each fermentation cycle, glucose was almost completely consumed within 24 h, while partial xylose remained unconsumed. The amount of unconsumed xylose increased from 4.2 to 10.4 g/L after six rounds of recycling (Figure 9). This result might be due to the decreased xylose consumption capacity of *Z. mobilis* 8b. DA pretreatment is known to generate more inhibitory degradation products compared to ammonia pretreatment (Lau et al., 2009), which might have caused less growth of OD and reduced fermentation performance with recycled cells. As reported by a previous study, 1 g diluted acid pretreated CS could release 24 mg furans, 4 mg aromatic compounds and 44 mg carboxylic acids, which was higher than that released from ammonia pretreated CS (Chundawat et al., 2010). Thus, the presence of more degradation product may pose significant stress to the cell growth. Because of the insufficient sugar consumption, ethanol titer decreased from 33.1 g/L for the first cycle to 30.3 g/L for the 6th cycle, while the metabolic yield was between 89.7 and 92.6%. The ethanol productivity was between 1.58 and 1.75 g L^–1^ h^–1^, which was much higher than 0.54 g L^–1^ h^–1^ for traditional fermentation (Figure 8A). Overall, high cell density fermentation with cell recycling worked for around five cycles in DA-CS hydrolysate without reducing ethanol titer compared to traditional fermentation. Together with the fermentation performance of A-CS hydrolysate, these results suggested that *Z. mobilis* 8b in A-CS hydrolysate could be recycled with good fermentation performance as compared to that in DA-CS hydrolysate.

## Conclusion

The fermentation performance of *Z. mobilis* 8b significantly depended on the composition/concentration of sugars, initial OD and especially nutrient concentration. Sufficient nutrient level seems necessary for satisfactory glucose and xylose co-fermentation. High cell density fermentation with cell recycling of *Z. mobilis* 8b worked well in YP medium and showed high ethanol productivity. Sugar consumption rates significantly decreased in lignocellulosic hydrolysates, which resulted in the decrease of ethanol productivity. High cell density fermentation with cell recycling greatly shortened fermentation time and improved ethanol productivity in both DA-CS and A-CS hydrolysate with increased ethanol titer and metabolic yield, while *Z. mobilis* 8b in A-CS hydrolysate showed higher cell recyclability.

## Data Availability Statement

All datasets generated for this study are included in the manuscript/supplementary files.

## Author Contributions

YL performed most of the experiments, data acquisition, and data interpretation, and wrote the manuscript. RZ supervised the execution of the experiments, analyzed the data, assisted in coordinating this study, and wrote the manuscript. XJ, XC, and XY performed the pretreatment of corn stover. ZL provided the technical assistance. MJ coordinated this study, designed the experiments, evaluated the data, and wrote the manuscript. All authors read and approved the final version of manuscript.

## Conflict of Interest

The authors declare that the research was conducted in the absence of any commercial or financial relationships that could be construed as a potential conflict of interest.
